# Efficacy and safety evaluation of adjuvant auricular acupuncture for smoking cessation: A study protocol of randomized, assessor-blinded, pragmatic pilot trial

**DOI:** 10.1097/MD.0000000000031456

**Published:** 2022-10-28

**Authors:** Won-Suk Sung, In Suh Choi, Jeong-Hyun Moon, Soo-Yeon Chae, Min-Gi Jo, Jung-Hyun Kim, Yeon-Cheol Park, Eun-Jung Kim, Yong-Hyeon Baek, Geun-Woo Kim, Byung-Kwan Seo

**Affiliations:** a Department of Acupuncture & Moxibustion, Dongguk University Bundang Oriental Hospital, Seongnam-si, Republic of Korea; b College of Korean Medicine, Kyung Hee University Graduate School, Seoul, Republic of Korea; c College of Korean Medicine, Dongguk University Graduate School, Seoul, Republic of Korea; d Department of Acupuncture and Moxibustion Medicine, Kyung Hee University Korean Medicine Hospital at Gangdong, Seoul, Republic of Korea; e Department of Acupuncture and Moxibustion Medicine, Kyung Hee University College of Korean Medicine, Kyung Hee University Hospital at Gangdong, Seoul, Republic of Korea; f Department of Neuropsychiatry, Dongguk University Bundang Oriental Hospital, Seongnam-si, Republic of Korea.

**Keywords:** adjuvant treatment, auricular acupuncture, nicotine replacement treatment, randomized controlled trial, smoking cessation

## Abstract

Smoking negatively impacts public health. There are several treatments to quit smoking, and nicotine replacement treatment (NRT) reportedly doubles the smoking cessation rate, with some limitations. Acupuncture is an alternative option with proven effects on smoking cessation. However, there has been no definite report that indicates the efficacy and safety of auricular acupuncture (AA) combined with NRT on smoking cessation.

**Methods:**

This is a randomized, assessor-blind, and pragmatic pilot study. We will recruit 40 participants who want to stop smoking and randomly allocate them into an NRT group and an NRT + AA group with a 1:1 ratio. Participants will receive NRT for 4 weeks and the NRT + AA group will receive additional AA treatment with 5 AA points (Shenmen (TF4), lung (CO14), throat (TF3), inner nose (TG4), and endocrine (CO18)) twice a week for 4 weeks. Follow-up will be conducted 1 and 3 months after intervention completion. The primary outcome will be tobacco consumption and abstinence rate determined by calculating the rate of change in cigarette use and a urine test. Secondary outcomes will be the quality of life (EuroQol-5D and visual analogue scale), nicotine dependence (Fagerstrom test for nicotine dependence), nicotine withdrawal (Minnesota nicotine withdrawal scale), physical effects, satisfaction, and safety measurement (adverse events).

**Results:**

We will investigate the efficacy and safety of AA combined with NRT treatment for smoking cessation.

**Conclusion:**

Our study will provide additional clinical evidence for AA as an adjuvant treatment for smoking cessation.

**Trial registration number:**

Clinical Research Information Service (registration number: KCT0007212).

## 1. Introduction

Smoking is a major preventable public health threat.^[1]^ It causes 6 million direct deaths annually and has induced approximately 900,000 deaths from secondhand smoke. Although not clarified, the dangers of third-hand smoke have been raised.^[2]^ Globally, 25% of men and 5% of women smoke daily as of 2015,^[3]^ and it is reported that smoking requires more inpatient care than alcohol and illegal drug use combined.^[4]^ Researchers have focused on the deleterious effects on cancer, fertility, and respiratory and musculoskeletal system disease.^[5–7]^ Smoking increases insulin resistance and further causes metabolic syndrome and diabetes.^[8]^

Many smokers try to quit smoking. Of those attempting to quit without any treatment, 60% start smoking again within 2 weeks and only 5% succeed.^[9]^ Therefore, various treatments are administered, such as the medications bupropion and varenicline, which have proven efficacy through several randomized controlled trials.^[10]^ Nicotine replacement treatment (NRT) has doubled the successful smoking cessation rate.^[11]^ It is absorbed through the oral or nasal mucosa (via gum, Rosen, tongue, inhaler, or spray) or the skin, which differs from other products in that they absorb nicotine slowly and passively.^[12]^ NRT reduces motivation for smoking, prevents the physiological and mental withdrawal symptoms that occur during the cessation period, and stimulates the nicotine receptors and the release of dopamine in the ventral cortex of the brain.^[13]^

However, these treatments have some side effects such as nausea, insomnia, and headaches.^[14]^ Some drugs exhibit side effects such as anxiety, cravings, tension, dry mouth, dizziness, and sedation when used during the smoking cessation process.^[15]^ Acupuncture is 1 alternative method for smoking cessation. Inserting a needle in a specific place can correct the disturbance of a force known as qi. Also, acupuncture stimulates the nerves and connective tissue located throughout the body.^[16]^

Stimulating the auricular acupuncture (AA) point greatly influences the limbic system of the brain. This mechanism is mediated through the trigeminal and vagus nerves and the cervical flexus.^[17]^ In addition, when a smoker experiences withdrawal during smoking cessation, stimulating the ear can minimize these effects by controlling the release of dopamine and serotonin.^[18]^ According to studies from the United States and Norway, 40% of smokers successfully quit through AA,^[19]^ indicating that this treatment has a positive effect on decreasing smoking desire.^[20]^ In 1 study, adjuvant AA has resulted in more significant smoking cessation effects and stability than NRT alone.^[21]^

Regardless of this research, no randomized controlled trials focus on the effect of AA combined with NRT on smoking cessation. This study will investigate the efficacy and safety of adjuvant AA treatment on smoking cessation.

## 2. Methods

### 2.1. Objectives

This pilot study aims to assess the efficacy and safety of AA treatment combined with NRT for smoking cessation comparing NRT alone.

### 2.2. Ethics approval and registration

This study complied with the Helsinki Declaration and was approved by the Institutional Review Board (IRB) of Dongguk University Bundang Oriental Hospital (DUBOH 2022-0005; final version 1.1 on April 7, 2022) and registered in the Clinical Research Information Service (CRIS, KCT0007212) on April 19, 2022.

### 2.3. Design

**Figure 1.**
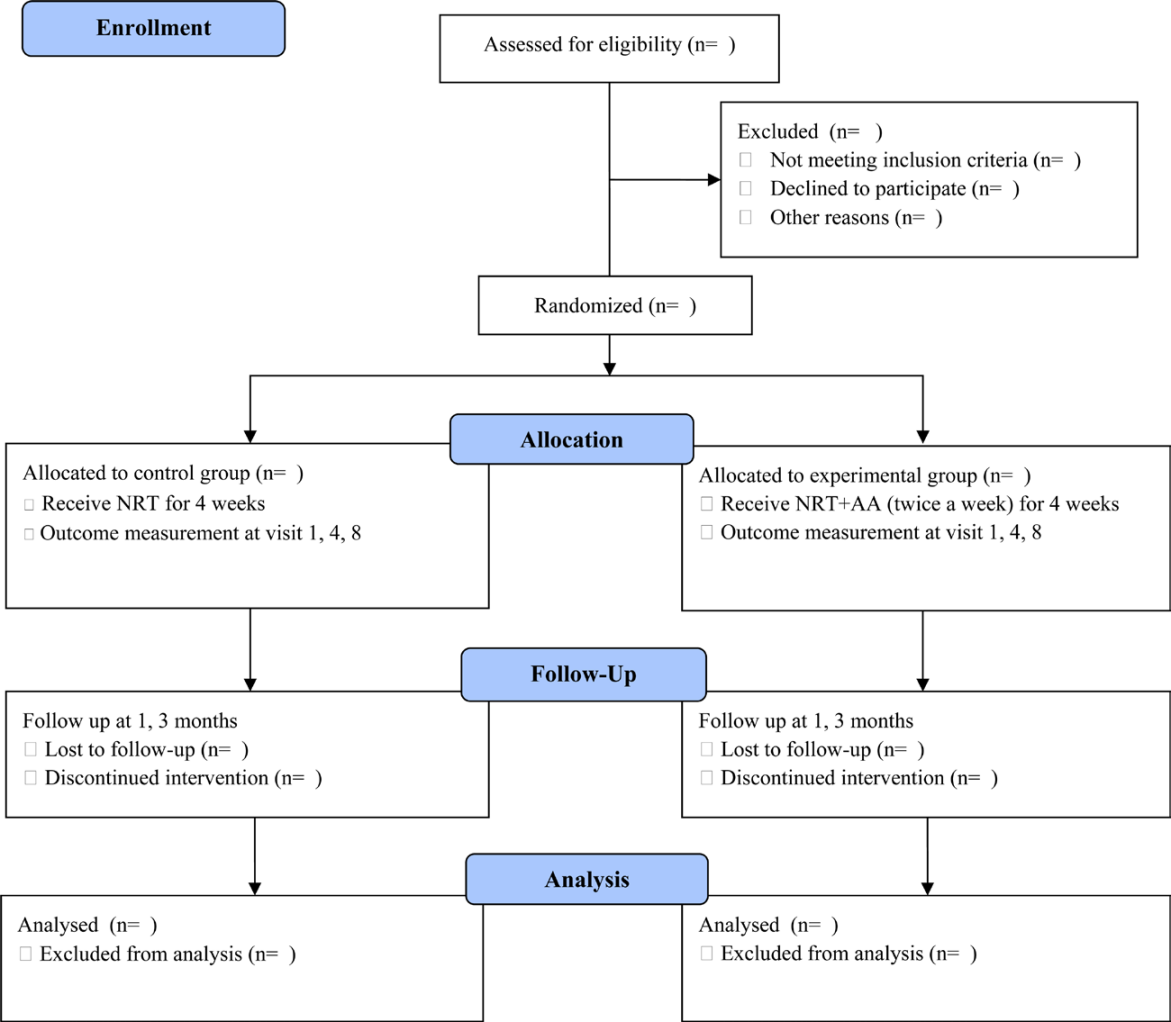
Flow diagram based on CONSORT 2010.^[22]^ AA = auricular acupuncture, NRT = nicotine replacement treatment.

This study is a randomized, assessor-blinded, pragmatic pilot clinical trial with 2 parallel groups. We intend to investigate the synergic effects of AA treatment on smoking cessation. Eligible participants will be assigned to 1 of 2 groups with a 1:1 ratio. The control group will receive only NRT, and the experimental group will receive AA treatment + NRT. The study period will include 4 week-treatment phase and 3 month-follow-up. The study design flowchart is as shown in Figure 1.

### 2.4. Participants

We will recruit 40 participants who wish to stop smoking with the following inclusion and exclusion criteria.

#### 2.4.1. Inclusion criteria.

We will include subjects who:

• are 19 to 70 years old;
• have no objection to nicotine patch and AA treatment;
• have been smoking more than 10 cigarettes a day;
• have attempted or are willing to quit smoking;
• use nicotine patches to quit smoking or are willing to receive assistance; and
• who voluntarily decided to participate in the clinical trial and provided written consent.

#### 2.4.2. Exclusion criteria.

We will exclude subjects who:

• have cardiovascular or lung diseases;
• struggle with AA, such as otitis externa;
• are reluctant to use acupuncture;
• have difficulty attaching nicotine patches and AA due to disease, including severe dermatitis;
• receive treatment for mental illness; or
• those who struggle with the questionnaire.

### 2.5. Sample size calculation

There have been several studies that investigated the additional efficacy of AA comparing conventional treatment (including NRT and counseling).^[21,23,24]^ However, these studies had some limitations such as using laser AA or a retrospective study. With these reasons, we could not quote fully by previous studies. According to Julious’s suggestion,^[25]^ we determined the appropriate sample size for a 2-parallel study was 12, and in consideration of the drop-out rate, we have decided to recruit 20 participants per group.

### 2.6. Recruitment

This study will be conducted in the Dongguk University Bundang Oriental Hospital. We will draft participants by recruitment posters, local newspapers, and public boards. The posters will contain information about the trial, inclusion/exclusion criteria, intervention methods and period expected benefits and risks, and the contact number.

### 2.7. Procedure

**Figure 2.**
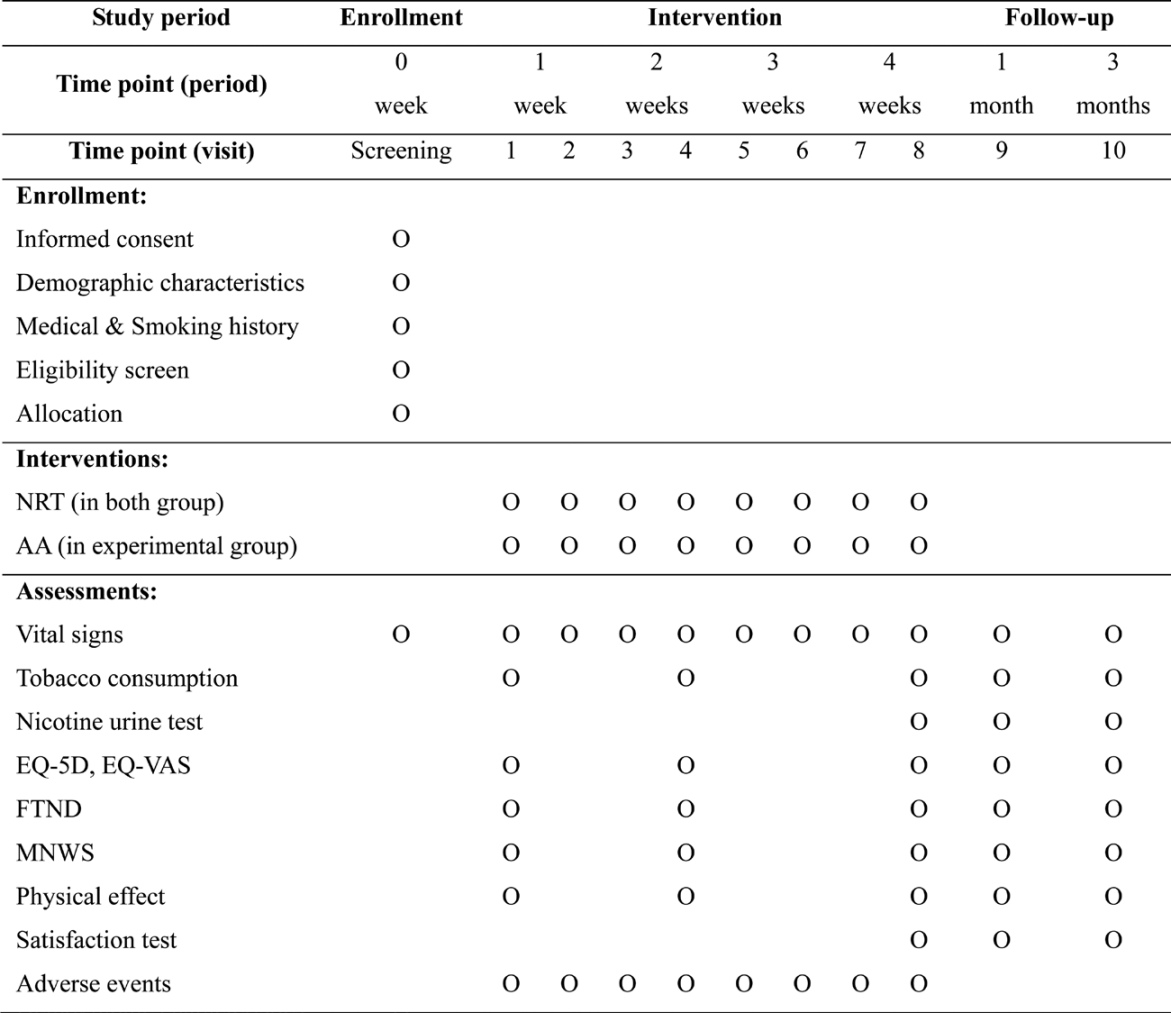
Study schedule. AA = auricular acupuncture, EQ = EuroQol, FTND = Fagerstrom test for nicotine dependence, MNWS = Minnesota nicotine withdrawal scale, NRT = nicotine replacement treatment, VAS = visual analogue scales.

When subjects interested in smoking cessation visit the hospital, a researcher will inform them of the study. This includes information regarding the study purpose, allocation ratio, intervention information (methods and period), expected benefits and risks, and other information required by the Korea Good Clinical Practice for the protection of subjects. Subjects will be informed that they can withdraw from the study at any time with no penalty. If they sign the informed consent form voluntarily, the investigator will collect participants’ demographic characteristics and medical and smoking history, check their vital signs, and determine their eligibility using the inclusion/exclusion criteria. Eligible participants will be assigned to the experimental or control group and undergo 8 treatments (twice weekly visits for 4 weeks, visit 1–8) and 2 follow-ups (1 and 3 months after intervention completion, visit 9–10) (Fig. 2).

### 2.8. Randomization and allocation concealment

A randomization sequence will be generated at a ratio of 1:1 by a statistician unrelated to this trial using R software (version 3.5.0; R Foundation for Statistical Computing, Vienna, Austria). The random code will be sealed in opaque envelopes and kept by the code manager. When a subject is eligible, researchers will open the envelope in front of the participant.

### 2.9. Blinding

The additional AA treatment necessitates that the participants and intervention practitioners cannot be blinded. Therefore, outcome assessors will be instructed to ask questions simply and write in detail to record case report form (CRF) and prevent them from conducting intervention to maintain the blinding.

### 2.10. Intervention

Participants in 2 groups will receive the following interventions. Other methods for smoking cessation such as medication will not be allowed during the study period.

#### 2.10.1. Control group intervention.

A nicotine patch named Nicotinell (manufactured by Lohmann Therapie-Systeme, Andernach, Germany) will be applied for 28 days. Each patch contains 52.5 mg of nicotine and participants will attach this patch and change new patch every day. If there is the patch that the participants have previously used, it will be allowed.

#### 2.10.2. Acupuncture intervention.

**Figure 3.**
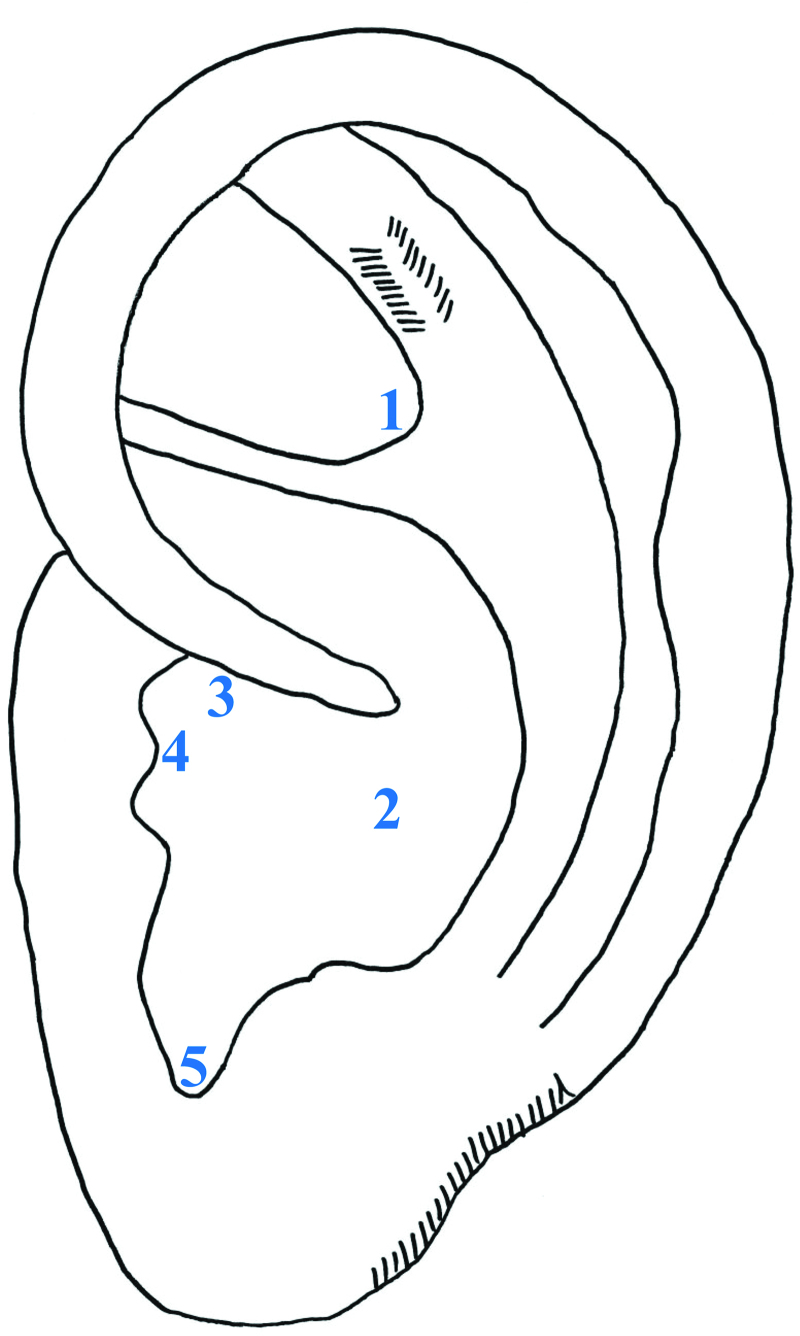
The 5 auricular acupuncture points in this study. 1 = shenmen (TF4), 2 = lung (CO14), 3 = throat (TF3), 4 = inner nose (TG4), 5 = endocrine (CO18).

Based on the control group intervention, participants will receive additional AA treatment. Ear press “T” needles (HL-03; Haeng Lim Seo Won Medical Co., Yeoju-gun, Gyeonggi-do, Republic of Korea) will be inserted at 5 AA points: the shenmen (TF4), lung (CO14), throat (TG3), inner nose (TG4), and endocrine (CO18) points (Table 1; Fig. 3). Previous research indicates that these points have a therapeutic effect.^[27–29]^ Participants will receive treatment twice a week for 4 weeks by a Korean medicine acupuncture and moxibustion specialist. For infection prevention, T needles will be inserted on the left ear in 1, 3, 5, 7 visit while others will be inserted on the right ear in 2, 4, 6, 8 visit.

**Table 1:** Detailed acupuncture treatment based on STRICTA 2010.^[26]^

Item	Detail	
1. Acupuncture rationale	1a) Style of acupuncture (e.g., Traditional Chinese Medicine, Japanese, Korean, Western medical, Five Element, ear acupuncture, etc)	Auricular acupuncture (Ear acupuncture)
	1b) Reasoning for treatment provided, based on historical context, literature sources, and/or consensus methods, with references where appropriate	Based on textbook of acupuncture and moxibustion and previous studies
	1c) Extent to which treatment was varied	All participants will receive nicotine replacement treatment commonly
2. Details of needling	2a) Number of needle insertions per subject per session (mean and range where relevant)	Control group: 0
		Experimental group: 5
	2b) Names (or location if no standard name) of points used (uni/bilateral)	Shenmen (TF4)
		Lung (CO14)
		Throat (TF3)
		Inner nose (TG4)
		Endocrine (CO18)
		Left side at visit 1, 3, 5, 7
		Right side at visit 2, 4, 6, 8
	2c) Depth of insertion, based on a specified unit of measurement, or on a particular tissue level	1mm
	2d) Response sought (e.g., *de qi* or muscle twitch response)	De-qi
	2e) Needle stimulation (e.g., manual, electrical)	T-needles are inserted
	2f) Needle retention time	2–3 d
	2g) Needle type (diameter, length, and manufacturer or material)	T-needles
3. Treatment regimen	3a) Number of treatment sessions	8 sessions
	3b) Frequency and duration of treatment sessions	Twice a week for 4 wk
4. Other components of treatment	4a) Details of other interventions administered to the acupuncture group (e.g., moxibustion, cupping, herbs, exercises, lifestyle advice)	Participants will receive nicotine patch and will be asked to attach 1 patch a day for 4 wk
	4b) Setting and context of treatment, including instructions to practitioners, and information and explanations to patients	Practitioners are instructed to prevent talking with participants
5. Practitioner background	5) Description of participating acupuncturists (qualification or professional affiliation, years in acupuncture practice, other relevant experience)	Korean medicine doctors who are specialist in acupuncture and moxibustion at least 7 yr of experience, or under supervision by a specialist
6. Control or comparator interventions	6a) Rationale for the control or comparator in the context of the research question, with sources that justify this choice	Previous studies about smoking cessation
	6b) Precise description of the control or comparator. If sham acupuncture or any other type of acupuncture-like control is used, provide details as for Items 1 to 3 above	Participants will receive nicotine patch and will be asked to attach 1 patch a day for 4 wk

### 2.11. Outcome measurement

#### 2.11.1. Tobacco consumption and abstinence rate.

The primary outcome will be reductions in daily cigarette smoking, measured in the self-reported number of cigarettes smoked per day. The survey will be completed during visit 1, 4, 8, 9, and 10. The abstinence rate will be determined by the rate of change in the number of cigarettes smoked between the first and last visits (visit 10). Smoking cessation will be biochemically validated by a urine test. The subjects will be asked to submit the urine sample at visit 8 to 10.

#### 2.11.2. Quality of life.

Quality of life will be measured by the EuroQol (EQ)-5D and the EQ-visual analogue scales (VAS) at visit 1, 4, 8, 9, and 10. EQ-5D is a generic measure of health status that can be used to clinically and economically evaluate health care and population health surveys.^[30]^ EQ-5D assesses health status in 5 dimensions: mobility, self-care, usual activities, pain and discomfort, anxiety, and depression. In EQ-VAS, participants will mark their overall daily health from 0 (worst) to 100 (best). In this study, higher scores will indicate a better quality of life.

#### 2.11.3. Nicotine dependence.

Nicotine dependence will be assessed using the Fagerstrom test for nicotine dependence at visit 1, 4, 8, 9, and 10. Fagerstrom test for nicotine dependence consists of 6 items that assess the period of heaviest smoking, including smoking when ill or smoking in places where it is prohibited.^[31]^ The 6 questions will be graded on a 2 to 4-point scale (0–1 to 0–3), which is summed to gain a total score (0–10). Higher scores indicate higher dependence.

#### 2.11.4. Nicotine withdrawal.

Nicotine withdrawal will be determined using the Minnesota nicotine withdrawal scale (MNWS), one of the most frequently used measures of nicotine withdrawal which generally reflect nicotine withdrawal syndrome symptoms in the DSM-IV.^[32]^ Each 9 withdrawal symptom (craving, irritability, anxiety, difficulty concentrating, restlessness, increased appetite or weight gain, depression, insomnia, and impatience) will be graded on a 5-point scale (1–5). Higher scores indicate a stronger nicotine withdrawal. The MNWS will be assessed at visit 1, 4, 8, 9, and 10.

#### 2.11.5. Physical effects.

The physical effects of smoking include irritability, tiredness, calmness, anxiety, cravings, unpleasant taste while smoking, headache, the ability to concentrate, and appetite. We will measure 4 domains that do not overlap with MNWS: tiredness, calmness, unpleasant taste while smoking, and headache, using a VAS from 0 to 100. Physical effects will be assessed at visit 1, 4, 8, 9, and 10.

#### 2.11.6. Satisfaction.

Satisfaction with NRT or NRT combined with AA treatment will be measured at the last treatment (visit 8) and during the follow-up phase (visit 9, 10).

#### 2.11.7. Safety measurement.

Adverse events will be assessed throughout the treatment period. Participants will be told to report information regarding adverse events and practitioners will follow up at regular or additional visits by several methods like clinical testing or history taking. All information, including the day of onset and disappearance, severance, relationship to the study, possible cause, and treatment, will be recorded.

### 2.12. Statistical analysis

We will express continuous variables as means ± standard deviation. For the statistical analysis, we will use paired *t* test for intra-group comparison and a 2-sample *t* test or Wilcoxon rank sum test for comparison between 2 groups. Categorical variables will be expressed as frequencies or percentages and tested statistically with the chi-squared test or Fisher’s exact test. All statistical analyses will be performed with the STATA version 15.0 (STATA Corp, LP. College Station, TX). Significance will be considered at a *P*-value < .05.

### 2.13. Date management and quality control

Data in this study will be collected in CRF by researcher in charge who are not related to intervention. For data confidence, documents related to study (consent from, CRF, other records) will be stored in locked space or a computer with password for 3 years after the study completion. To conduct clinical research smoothly and guarantee the quality of the research, all researchers will receive the pre-education and participate the initiation meeting before the research initiation.

### 2.14. Data monitoring

There is no need for data monitoring committee because NRT and AA are commonly used and minimally invasive interventions, but data quality will be maintained by the IRB’s regular monitoring. IRB will check whether there is an audit or study-related modification and determine the continuation of the study in a critical situation. This process will ensure the scientific validity of this study and the protection of the rights of participants.

## 3. Discussion

Since acupuncture was first used for smoking cessation in 1977,^[33]^ it has been recognized for its simple operation and low cost.^[34]^ Recent studies have shown that AA is an alternative option.^[21]^ This study will clarify the efficacy and safety of AA combined with NRT for smoking cessation and suggest clinical evidence to clinicians and policymakers in their decision-making.

## Author contributions

**Conceptualization:** Eun-Jung Kim, Won-Suk Sung.

**Funding acquisition:** Byung-Kwan Seo.

**Investigation:** In Suh Choi, Jeong-Hyun Moon, Soo-Yeon Chae, Min-gi Jo, Jung-Hyun Kim.

**Methodology:** Eun-Jung Kim, Geun-Woo Kim.

**Project administration:** Won-Suk Sung, Byung-Kwan Seo.

**Supervision:** Byung-Kwan Seo.

**Writing – original draft:** In Suh Choi, Won-Suk Sung.

**Writing – review & editing:** Eun-Jung Kim, Yeon-Cheol Park, Yong-Hyeon Baek, Geun-Woo Kim.
